# Comparative Evaluation of Antimicrobial Activity of Cotton Balls Incorporated With Musa paradisiaca and Chitosan: An In Vitro Study

**DOI:** 10.7759/cureus.27553

**Published:** 2022-08-01

**Authors:** Keerthanasri Subramaniyan, Umesh Kemparaj, Sangeeta Chavan, Muthu Karuppaiah, Palanivel Pandian

**Affiliations:** 1 Public Health Dentistry, Best Dental Science College, Madurai, IND

**Keywords:** gentamicin, antimicrobial activity, soluble chitosan, banana peel extract, cotton balls

## Abstract

Introduction: Antibiotics are one of the great advances in medicine. But overusing them has led to resistant bacteria (bacteria that are harder to treat). The current study foresees better non-toxic antimicrobial substances (conventional antibiotics) that insist to consider medicinal plants and animal-derived products, which have better antibiotics without any side effects. The goal of this in-vitro study was to evaluate the antimicrobial activity of cotton balls incorporated with *Musa paradisiaca* and chitosan.

Materials and methods: *Musa paradisiaca*, chitosan, and gentamicin-reinforced cotton balls were considered in three groups namely Group 1, Group 2, and Group 3, which tested against the strains of *Staphylococcus aureus, Escherichia coli*, *Actinomyces israelii, Streptococcus mutans, *and*Bacteroides fragilis*. For the present study, pre-sterilized cotton balls were taken and then soaked with Banana peel extract and soluble chitosan solution at different concentrations of 500 μg/ml, 250 μg/ml, 100 μg/ml, and 50 μg/ml under aseptic conditions and were dried at 50° overnight. The same incorporation method was followed for a 10mg/ml concentration of gentamicin, which was used as a positive control group.

Results: In this current study, the banana peel extract, soluble chitosan, and gentamicin exhibited antimicrobial activity against all the tested microorganisms. In the well diffusion method, at the concentration of 500 μg/ml and 250 μg/ml, chitosan and banana peel extract were comparatively better than the positive control group (gentamicin) at a higher concentration of 10 mg/ml.

Conclusion: From the results of the present study, a lower concentration of the testing group (soluble chitosan and banana peel extract) exhibited a better effect when compared to a higher concentration of gentamicin. Hence, chitosan and banana peel extract impregnated cotton could be preferred for routine clinical scenarios like wounds, extractions sockets, and during any short intraoperative surgical procedures periodontal surgery, where it can provide maximal antimicrobial effects without the side effects of antibiotics.

## Introduction

Microbial infectious diseases are considered the major etiological cause of 50,000 deaths per day worldwide [1]. Especially, the oral cavity is found to harbor a huge community of microorganisms that is found within various soft and hard tissues like tooth, tongue, hard palate, and gingiva associated with its biofilm.

However, they were also been considered as the causative agents for several other systemic diseases. It is necessary to consider the antimicrobial resistance of many bacteria present in the oral biofilm at concentrations 1,000 to 1,500 times higher than the commonly used concentration. In recent years, many disadvantages of commercial antibiotics such as ampicillin, chlorhexidine, quaternary ammonium compounds, and metronidazole were discovered that including drug resistance to pathogenic bacteria [2], associated with adverse effects such as hypersensitivity, immunosuppression, and allergic reactions, tooth staining, diarrhea, increased calculus formation, and changes in the bowel flora [3], has led to a search for better non-toxic antimicrobial substances that insist to consider medicinal plants that have better antibiotic effects without any side effects [4,5].

Plants with medicinal properties are studied from ancient times as it possesses many beneficial characteristics to mankind and their compounds form the basic foundation of today’s modern prescription drugs [6]. One such plant includes *Musa paradisiaca*. *Musa paradisiaca *L. belongs to the Musaceae family commonly called a banana plant whose fruit, stem juice, and flowers were traditionally used to treat disease in various forms. Pharmacological investigation on this reveals its antioxidant activity, anti-diabetic, wound healing activity, and antibiotic [7].

Chitosan is a deacetylated derivative of polysaccharide chitin, present in exoskeletons of insects and cell walls of fungi mainly in the order Mucorales. It possesses peculiar properties like excellent biocompatibility, nontoxicity, increased bioactivity, good biodegradability, better reactivity of the group amino deacetylated, selective permeability of substance, polyelectrolyte activity, antimicrobial activity, gelling ability, chelating action, and absorptive nature [8] that lead to a wide range of applications such as drug carrier with the controlled release [9], anti-bacterial agent, and antacid. Thus, it inhibits the oral bacterial plaque formation and prevents decalcification of dental enamel [10,11], promotes osteogenesis, and absorbs fat from adjacent tissue promoting healing. The aim of this in vitro study is to compare and evaluate the antimicrobial activity of cotton balls incorporated with *Musa paradisiaca* (banana peel extract) and chitosan (soluble chitosan).

## Materials and methods

An in vitro study was conducted to determine the antimicrobial efficacy of *Musa paradisiaca *(Banana peel extract) and chitosan (soluble chitosan) incorporated in cotton balls. The antimicrobial efficacy of the study samples was assessed against the strains of *Staphylococcus aureus, Escherichia coli, Actinomyces israelii, Streptococcus mutans, *and *Bacteroides fragilis*. These strains were obtained from the American Type Culture Collection (ATCC), Chandigarh, India. The study product (*M. paradisica* and chitosan incorporated cotton balls) was prepared and tested against organisms at Trichy Research Institute of Biotechnology (P) Ltd, Tiruchirappalli, Tamil Nadu, India.

Extract preparation from the banana peel

**Figure 1:**
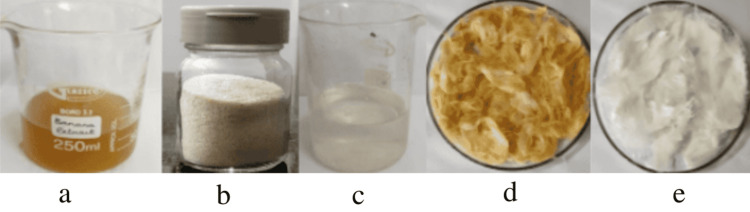
Steps for incorporation a) Banana peel extract, b) Chitosan powder, c) Soluble chitosan, d) Incorporation of banana peel extract, e) Incorporation of soluble chitosan

The fresh banana was sourced from the local market at Pudukkottai, Tamil Nadu, India, and the peels were washed with distilled water to remove impurities and then dried under sun/shade for four days. After drying, it was pulverized and stored in a sterile container. A quantity of 10 gm of banana powder was mixed with 100 ml of isopropanol and incubated at 4^o^C for 24 hours. Following incubation, the immersed material was separated using muslin cloth followed by filtration with Whatman™ Grade 1 paper (Whatman PLC, Maidstone, United Kingdom). The solution was evaporated until dry matter was obtained at the end of the procedure. A varied concentration of 500 μg/ml, 250 μg/ml,100 μg/ml, and 50 μg/ml banana extract solution was prepared by adding distilled water (Figure 1a).

Preparation of soluble chitosan

**Figure 2:**
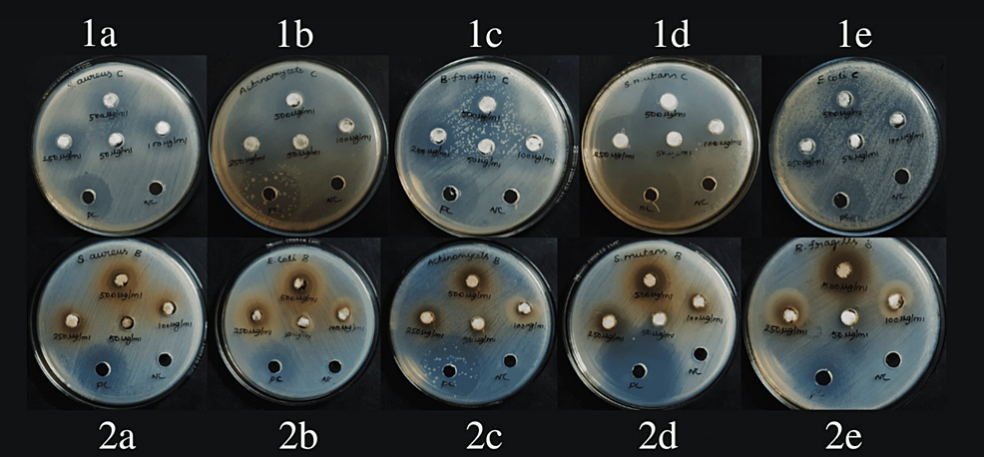
Measurement of the zone of inhibition Measurement of the zone of inhibition with banana peel extract: (1a) *Staphylococcus aureus; *(1b)  *Actinomyces israelii; *(1c) *Bacteroids fragilis; *(1d) *Streptococcus mutans; *(1e) *E.Coli* Measurement of the zone of inhibition with soluble chitosan: (2a) *Staphylococcus aureus; (*2b) *E.Coli; *(2c*) Actinomyces israelii; *(2d) *Streptococcus mutans; *(2e) *Bacteroids fragilis*

About 1 gm of chitosan was obtained from Swakit Biotech Private Limited, Bengaluru, Karnataka (Figure 2b). It was mixed with 200 µl of glacial acetic acid and dissolved in 100 ml of double-distilled water and stirred continuously for one hour using a magnetic stirrer. The solution was evaporated to get a 100% concentration solution. Different concentrations of study material (soluble chitosan and banana peel extract) were made using serial dilution [12] (Figure 1c).

Incorporation of banana peel extract and soluble chitosan on cotton balls

For the present study, pre-sterilized cotton balls were taken and then soaked with banana peel extract and soluble chitosan solution at different concentrations of 500 μg/ml, 250 μg/ml, 100 μg/ml, and 50 μg/ml under aseptic conditions and were dried at 50° overnight (Figures 1d, 1e). The same methods and 10mg/ml concentration of gentamicin are used as a positive control group.

Laboratory estimation procedure

Soluble chitosan and banana peel extract were used as a test group named Group C and Group B. The petri plates containing 20 ml nutrient agar medium were seeded with a 24-hour culture of bacterial strains (*S. aureus, E.Coli, A. israelii, B. fragilis*, and *S. mutans*), which were adjusted to 0.5 OD value according to McFarland standard. Wells were cut and different concentrations of 500, 250, 100, and 50 μg/ml were added for test groups and incubated at 37°C for 24 hours.

The antimicrobials of the sample were allowed to diffuse into the medium with test organisms. The resulting zone of inhibition that is uniformly circular because of a confluent lawn of growth is used for the analysis of antibacterial activity using the assay. The diameters of the inhibition zone formed around the wells were considered (Figure 2). The antibiotic gentamicin was the positive control group. The data were calculated using GraphPad Prism version 6.0 for Windows (GraphPad Software, San Diego, California, United States).

The data analysis was performed using IBM SPSS Statistics for Windows, Version 26.0 (Released 2019; IBM Corp., Armonk, New York, United States).

## Results

The concentrations of 50 μg/ml and 100 μg/ml of soluble chitosan have a larger zone of inhibition when compared to banana peel extract with all organisms except *E.coli* (Table 1). At the maximum tested concentration, soluble chitosan was comparatively better with three organisms (*S.aureus*, *A.israelii*, and *S.mutans*) with a maximum zone of inhibition. At 250 μg/ml and 500 μg/ml of chitosan (Group C), a larger zone of inhibition is seen against *S.aureus, A. israelii, *and *S.mutans* (19.75, 17.75 and 19.75, respectively) (Table 1). As compared to the banana peel extract group against *E.coli and B.fragilis*, the soluble chitosan group has shown a larger zone of inhibition. The zone of inhibition observed against *S.mutans* was higher compared to the positive control group by chitosan impregnated cotton balls.

**Table 1:** Descriptive statistics on zones of inhibition obtained by banana peel extract and soluble chitosan at varied concentrations against specific microorganisms. B: banana peel extract; C: soluble chitosan; PC: positive control group (gentamicin)

S. No	Name of the test organism	Maximum zone of inhibition (mm)									
		500 μg/ml		250 μg/ml		100 μg/ml		50 μg/ml		PC (10mg/ml) (Gentamicin)	
		B	C	B	C	B	C	B	C	B	C
1	Staphylococcus aureus	13.25	19.75	9.75	15.2	0	11.15	0	8.1	21.5	19.5
2	Escherichia coli	26	12.75	23.5	11.25	12.75	8.7	8.15	7.25	21.5	21.5
3	Actinomyces israelii	11	17.75	9.45	13.75	7.25	11.75	0	9.75	23.5	19.5
4	Streptococcus mutans	11.5	19.75	11.35	19.2	7.25	17.15	6.2	12.1	24.5	18.7
5	Bacteroides fragilis	18	11.75	12.75	10.25	8.2	8.2	6.15	7.15	19.5	18.7

All the tested microorganisms showed antimicrobial activity. In the well diffusion method, at the minimal concentration of 500 and 250 μg/ml, soluble chitosan and banana peel extract were comparatively better than the positive control group (gentamicin) at a higher concentration of 10 mg/ml. In a comparison of soluble chitosan and banana peel extract, soluble chitosan has a better effect (Table 1).

## Discussion

The current study was conducted to evaluate the antimicrobial efficacy of cotton balls impregnated with *Musa paradisiaca* and chitosan. The microorganisms used in this study are *S. aureus, E. Coli, A. israelii, B. fragilis, *and* S. mutans*. The application is not limited to the oral cavity alone and can be applied to all traumatic and surgical procedures. The antimicrobial efficacy was tested by diffusing out the study material into the medium and interacting in a plate freshly seeded with the test organisms.

In the present study, chitosan has been shown to have antimicrobial action against pathogenic organisms even at a low concentration (50 μg/ml and 100 μg/ml) and its effect at a concentration of 500 μg/ml was larger than the positive control group. An in vitro study conducted by Sarkar et al. in 2020 regarding the antimicrobial efficacy of chitosan on various periodontal pathogens reported a positive result [8]^_. _^Another study by Imani et al. in 2018 showed that chitosan has antibacterial activity against *Enterococcus‎ faecalis, S. aureus, *and *S.‎ mutans *[9]_._

The present study showed that at 500 μg/ml, chitosan has a larger zone of inhibition (19.75) against *S. aureus *and *S. mutans*. Chung et al. studied the antibacterial effect of water-soluble chitosan on representative dental pathogens such as *S. Mutans *and* Lactobacilli brevis* and concluded that chitosan has a better effect [10]. The mechanism of antimicrobial action of chitosan might be that it has positively charged reactive amino groups, which interact with the negatively charged bacterial cell wall, resulting in the leakage of intracellular substances leading to the death of microorganisms [13]_._

In this present in vitro study, the results followed literature evidence that banana extract has a considerable effect on pathogens like *E.Coli *and *B.fragilis* in comparison with chitosan or gentamicin control group and also shows some effects against other antimicrobial pathogens. 

The banana peel, which is the outer shell of a banana, has lots of beneficial effects like relieving pain, reducing swelling, and anti-itching, whether it is used alone or combined with ointments or cream-based. Also, it possesses multiple effects like antibacterial, antihypertensive, and anti-inflammatory. Although the banana peel contains high phenolic content and fatty acid, which possesses antimicrobial activity, in this current study, the banana powder was mixed with 75% of isopropyl alcohol, which acts for dehydration purposes.

An in vitro study by Kavitha et al. in 2019 evaluated the antibacterial activity of both fresh and dried various banana peel extracts against the pathogens* S. aureus, Bacillus subtilis, *and *Pseudomonas aeruginosa* and concluded that alcoholic peel extracts of fresh and dried bananas could be considered as a good antibacterial agent against both Gram-positive and Gram-negative bacteria [14].

Hassan et al. conducted an in vitro and in vivo study regarding the anti-toxicity of banana peel extract [15], The antimicrobial activity of banana peel extracts was tested against some pathogenic bacteria and showed the capacity to have a broad range of inhibition activities against* E. coli, S. aureus, *and* P. aeruginosa*.

Limitations in the current study for clinical reproduction are that other biological parameters influencing microbial count could not be considered; thus, an in vivo study would better emphasize clinical reproduction.

## Conclusions

From the results of the present study, a lower concentration of the testing group (soluble chitosan and banana peel extract) has a better effect when compared to a higher concentration of gentamicin. Hence, chitosan and banana peel extract impregnated cotton could be preferred for routine clinical scenarios like wounds, extractions sockets, and during any short intraoperative surgical procedures periodontal surgery, where it can provide maximal antimicrobial effects without the side effects of antibiotics.
